# Ethanol Electrooxidation at 1–2 nm AuPd Nanoparticles

**DOI:** 10.3390/nano12224093

**Published:** 2022-11-21

**Authors:** Juliette W. Strasser, Richard M. Crooks

**Affiliations:** Department of Chemistry, Texas Materials Institute, The University of Texas at Austin, 2506 Speedway, Stop A5300, Austin, TX 78712-1224, USA

**Keywords:** electrocatalysis, ethanol electrooxidation, alloy nanoparticles, gold nanoparticles, palladium nanoparticles, scanning transmission electron microscopy, energy dispersive spectroscopy

## Abstract

We report a systematic study of the electrocatalytic properties and stability of a series of 1–2 nm Au, Pd, and AuPd alloy nanoparticles (NPs) for the ethanol oxidation reaction (EOR). Following EOR electrocatalysis, NP sizes and compositions were characterized using aberration-corrected scanning transmission electron microscopy (ac-STEM) and energy dispersive spectroscopy (EDS). Two main findings emerge from this study. First, alloyed AuPd NPs exhibit enhanced electrocatalytic EOR activity compared to either monometallic Au or Pd NPs. Specifically, NPs having a 3:1 ratio of Au:Pd exhibit an ~8-fold increase in peak current density compared to Pd NPs, with an onset potential shifted ~200 mV more to the negative compared to Au NPs. Second, the size and composition of AuPd alloy NPs do not (within experimental error) change following 1.0 or 2.0 h chronoamperometry experiments, while monometallic Au NPs increase in size from 2 to 5 nm under the same conditions. Notably, this report demonstrates the importance of post-catalytic ac-STEM/EDS characterization for fully evaluating NP activity and stability, especially for 1–2 nm NPs that may change in size or structure during electrocatalysis.

## 1. Introduction

We report a systematic study of electrochemical ethanol oxidation reaction (EOR) activity at 1–2 nm AuPd dendrimer-encapsulated nanoparticles (DENs) [1,2,3,4], with particular attention focused on the stability of the nanoparticles (NPs). Two conclusions emerge from this study. First, alloyed AuPd DENs exhibit improved electrocatalytic EOR activity compared to either monometallic Au or Pd DENs. Specifically, DENs having a 1:1 or 3:1 ratio of Au:Pd exhibit higher peak current densities compared to Pd DENs, and more negative onset potentials compared to Au DENs. Second, chronoamperometry experiments, 1.0 or 2.0 h in length, result in growth of monometallic Au DENs, but the size and composition of AuPd alloy DENs are stable. Notably, this study demonstrates the importance of post-catalytic microscopy when evaluating EOR activity and stability, especially for 1–2 nm NPs.

AuPd NP catalysts have previously been shown to have enhanced activity for the electrocatalytic EOR compared to monometallic Au or Pd NPs [5,6,7,8,9,10,11,12]. In one study of 3–5 nm AuPd NPs, for example, EOR activity was reported to improve compared to that of similarly sized Pd-only NPs [8]. Specifically, increased catalytic current density and resistance to poisoning were observed at a 3:1 Au:Pd alloy. In a similar study, the EOR activity of a 10 nm AuPd alloy reached a maximum for a 1:1 ratio of Au:Pd [9]. In both cases, the authors proposed that alloying Au with Pd resulted in decreased binding of reaction intermediates that could poison the NP surface, thereby improving the EOR activity. Taken together, these studies suggest that alloying Au with Pd is an effective means for tuning the binding energies of the reaction intermediates, and consequently improving the catalytic activity of NPs.

When evaluating the electrocatalytic activity of NP catalysts, the degree of catalyst stability is important. For the electrocatalytic EOR, catalyst stability is usually assessed using chronoamperometry [5,8,9]. Specifically, the potential of the electrode is typically held at the peak potential of the electrocatalytic cyclic voltammogram (CV) for times ranging from 30 min to 12 h. Significantly, chronoamperometric stability measurements do not provide insight into size or structural changes of the catalysts under investigation. Recently, Peng et al. used identical location transmission electron microscopy (IL-TEM) to assess the stability of 5 nm Pd NPs following a 1 h chronoamperogram (CA) under EOR conditions [13]. The IL-TEM indicated that continuous EOR induced NP aggregation and Pd dissolution, thereby demonstrating that long-term electrocatalytic studies can significantly change the size and composition of the catalysts under study.

The size and structure of NPs both before and after electrochemical measurements can be characterized using techniques such as scanning transmission electron microscopy (STEM) and energy dispersive spectroscopy (EDS) [14,15]. For example, our group used STEM to show that AuNPs grow from 2 to 6 nm during the electrochemical CO_2_ reduction reaction [16]. Transmission electron microscopy (TEM) was also used to demonstrate that 1–2 nm AuNPs grow to a limiting size of ~2 nm as a result of electrochemical cleaning scans [17]. Additionally, it has been shown that 2–6 nm Pd NPs rapidly degrade during potential cycling in acid, which leads to a loss of HER activity for the catalyst [18]. Similarly, it has been reported that a 60% loss of electrochemically active surface area occurs for 2–5 nm Pt NPs upon potential cycling in alkaline solutions [19]. In all cases, electrochemical measurements led to changes in NP size, which often corresponded to significant changes in the electrocatalytic activity of the NPs. Alloy NPs may also undergo additional structural or chemical changes such as dealloying or atomic rearrangement, which can be characterized using EDS [20,21,22]. Changes in catalyst size and structure during electrochemical measurements are particularly problematic when experimental measurements are correlated to theory [23]. Consequently, it is important to carry out post-catalytic physical characterization of NPs when reporting electrocatalytic activity.

In the present report, monometallic 1–2 nm Au and Pd DENs and a series of 1–2 nm DENs having 3:1, 1:1, and 1:3 ratios of Au:Pd (denoted as G6-OH(Au_x_Pd_y_)_309_) in the same size range were prepared using a dendrimer templating method [24]. DENs were evaluated for their electrocatalytic EOR activity using cyclic voltammetry and chronoamperometry. The results showed that, compared to G6-OH(Pd_309_) DENs, an ~8-fold increase in peak current density is observed for G6-OH(Au_3_Pd)_309_ DENs. Moreover, the onset potential is shifted ~200 mV more to the negative compared to G6-OH(Au_309_) DENs. Finally, and most importantly, post-catalytic STEM and EDS characterization reveal that G6-OH(Au_x_Pd_y_)_309_ alloy DENs retain their initial size and composition after 1.0 or 2.0 h of continuous EOR. In contrast, monometallic Au DENs aggregate under the same conditions.

## 2. Materials and Methods

### 2.1. Chemicals and Materials 

All chemicals were used as received unless otherwise noted. Sixth-generation poly(amidoamine) (PAMAM) dendrimers terminated with hydroxyl groups (G6-OH) were purchased from Dendritech Inc. (Midland, MI, USA). Dendrimers were purchased as a 10–15% solution in methanol. The methanol was removed under vacuum before use, and then the dendrimers were resuspended in water at a concentration of 100 µM prior to DEN synthesis. HAuCl_4_·3H_2_O (≥99.9%), K_2_PdCl_4_ (98%), NaBH_4_ (99.99%), Nafion 117 solution (5.0 wt% in methanol), and 1.0 M KOH and NaOH solutions were purchased from MilliporeSigma (Burlington, MA, USA). Ethanol (200 proof, 99.5+%), isopropanol (99.9%), and HPLC-grade water were purchased from ThermoFisher Scientific (Waltham, MA, USA). Vulcan carbon (XC-72R) was purchased from Fuel Cell Store (College Station, TX, USA). N_2_ (99.9999%) was purchased from Linde (Austin, TX, USA).

### 2.2. Synthesis of Au and Pd DENs

Dendrimer-encapsulated Au or Pd NPs having an average of 309 atoms per NP were synthesized according to previously published literature reports [16,24,25,26,27]. Dendrimers are highly branched, spherical polymers that can be used to template and stabilize NPs [1,28,29]. The resulting DENs are sterically confined to the interior cavity of the dendrimer, with only minimal interaction between the dendrimer and the NP surface [2,30,31]. Note that the stoichiometry of the DENs (e.g., G6-OH(Au_309_)) represents the HAuCl_4_:dendrimer ratio used during synthesis, and does not necessarily correspond to a precise NP structure. We have previously reported, however, that this ratio controls the average size of DENs and that the size distribution is narrow [2,32]. These comments also apply to Pd and Au_x_Pd_y_ DENs.

For the Au DEN synthesis, 309 equiv. of a 20 mM HAuCl_4_ solution were added dropwise to a stirred solution of 2.0 µM G6-OH dendrimer. After 2 min, an 11-fold excess of NaBH_4_ in 0.30 M NaOH was added to the stirred solution. The mixture was stirred for ~12 h in air at 22 ± 2 °C to deactivate excess BH_4_-. The final concentration of G6-OH(Au_309_) DENs was 2.0 µM.

For the Pd DEN synthesis, 309 equiv. of 20 mM K_2_PdCl_4_ were added dropwise to a 2.0 µM G6-OH solution and stirred for 30 min under N_2_ to allow for complexation of the Pd^2+^ precursor with the dendrimer [26]. After 30 min, an 11-fold excess of NaBH_4_ dissolved in water was added to the stirred solution. The final G6-OH(Pd_309_) concentration was 2.0 µM.

### 2.3. Synthesis of G6-OH(Au_x_Pd_y_)_309_ DENs

G6-OH(Au_x_Pd_y_)_309_ DENs were also prepared according to literature procedures [33,34]. Here, x and y are used to describe the ratio of Au:Pd added during DEN synthesis (e.g., G6-OH(AuPd)_309_ denotes a DEN having a 1:1 ratio of Au:Pd).

Using the synthesis of G6-OH(AuPd)_309_ DENs as an example, 154.5 equiv. of 20 mM K_2_PdCl_4_ solution were added to a 2.0 µM solution of G6-OH dendrimer and stirred for 30 min under N_2_. After 30 min, 154.5 equiv. of a 20 mM HAuCl_4_ solution were added dropwise to the stirred solution. Following an additional 2 min of stirring, an 11-fold excess of NaBH_4_ was added to the solution. This method was also used to prepare G6-OH(Au_3_Pd)_309_ and G6-OH(AuPd_3_)_309_ DENs. In these cases, the Au:Pd ratio was modified accordingly while keeping the total metal ion concentration constant. The final G6-OH(Au_x_Pd_y_)_309_ concentration in solution was 2.0 µM.

### 2.4. Electrode Preparation

Conductive inks containing DENs were prepared by first sonicating 200 µL of isopropanol, 10.0 µL of 5.0 wt% Nafion 117 (in methanol), and 1.0 mg of Vulcan carbon for 5–10 min. Next, 1.0 mL of a 2.0 µM DEN solution was added to the slurry and sonicated for an additional 5–10 min. Catalyst-coated electrodes were prepared by dropcasting 6.0 µL of the conductive ink on a 3.0 mm glassy carbon electrode (GCE) and drying under N_2_. Between each set of electrochemical measurements, the GCE was polished using a 0.05 µm alumina slurry.

### 2.5. Electrochemical Measurements

Electrochemical measurements were collected using a CH Instruments Model 650C Electrochemical Analyzer (Austin, TX, USA). The GCEs and the reference electrode, Hg/Hg_2_SO_4_, were purchased from CH Instruments (Austin, TX, USA). The counter electrode was a glassy carbon rod purchased from ThermoFisher Scientific (Waltham, MA, USA).

The electrocatalytic EOR measurements were carried out as follows. First, three background CVs were collected at a scan rate of 50 mV/s in a N_2_-satd., 1.0 M KOH solution. After the background CVs, ethanol was added to the solution, and N_2_ was bubbled through for an additional 5 min. Next, three electrocatalytic CVs were collected at a scan rate of 50 mV/s in N_2_-satd., 1.0 M KOH solution containing 0.50 M ethanol. The potentials used for collecting the CVs depended on the catalyst being analyzed, but were between 0 and −1.4 V.

For the cases where DENs were analyzed by ac-STEM and EDS after the CVs, the catalyst-coated GCE was removed from solution, gently rinsed, and used to prepare a TEM grid. Otherwise, the electrocatalytic CAs were carried out next using the same electrode. For CAs, the potential was held for 1.0 or 2.0 h at the peak potential of the CV. The CA solutions were not stirred unless otherwise indicated. Following the CA, three additional CVs were collected in the same solution. Finally, the catalyst-coated GCE was removed from solution, gently rinsed, and used to prepare a TEM grid.

### 2.6. ac-STEM and EDS Characterization

For ac-STEM and EDS, lacey carbon-coated, 400 mesh Cu TEM grids were purchased from Electron Microscopy Sciences (Hatfield, PA, USA). Two types of samples were analyzed. For the as-prepared catalyst inks, 0.5 µL of conductive ink containing DENs was diluted with 2.0 µL of H_2_O, and the resulting solution was dropcasted onto the lacey carbon-coated TEM grid. Following electrochemical analysis, TEM grids were prepared by gently wiping the lacey carbon-coated TEM grid across the wetted surface of the GCE. In both cases, the TEM grids were dried in a glove bag under N_2_.

ac-STEM and EDS analysis were carried out using a JEOL Neoarm ac-STEM (JEOL USA, Inc., Peabody, MA, USA) at an accelerating voltage of 80 keV and a resolution of 0.11 nm. For NP size analysis, ImageJ was used to evaluate 200 randomly selected NPs from each sample. The data were fit using the Gaussian function in the OriginLab software package (Version E8, Northampton, MA, USA). EDS data for each sample were collected and analyzed using the Pathfinder software package from ThermoFisher Scientific (Version 2.8, Waltham, MA, USA).

## 3. Results and Discussion

### 3.1. Preparation and Characterization of DENs

As described in the Experimental Section, G6-OH dendrimers were used as templates to prepare G6-OH(Au_309_), G6-OH(Pd_309_), G6-OH(Au_3_Pd)_309_, G6-OH(AuPd)_309_, and G6-OH(AuPd_3_)_309_ DENs. Vulcan carbon and Nafion binder were then used to prepare conductive inks containing DENs, which is also described in the Experimental Section [16,24].

**Figure 1:**
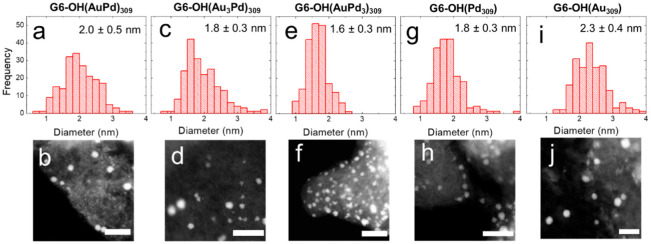
Size-distribution histograms and representative ac-STEM micrographs for conductive inks containing as-prepared (**a**,**b**) G6-OH(AuPd)_309_; (**c**,**d**) G6-OH(Au_3_Pd)_309_; (**e**,**f**) G6-OH(AuPd_3_)_309_; (**g**,**h**) G6-OH(Pd_309_); and (**i**,**j**) G6-OH(Au_309_) DENs. The scale bar is 10.0 nm.

Figure 1 shows ac-STEM micrographs and size-distribution histograms, obtained by sizing 200 randomly selected NPs, for the as-prepared DEN inks. 

Quantitative information about the distributions is shown in Table 1. The sizes provided in the table are consistent with previous reports [24]. The key point is that, within error, all of the as-prepared DEN compositions display the same initial average size.

**Figure 2:**
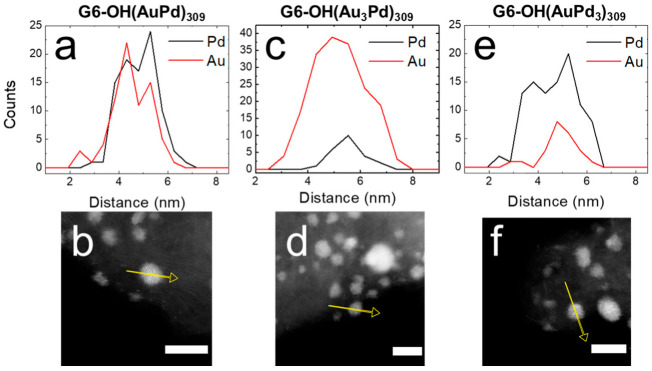
EDS line scans and corresponding ac-STEM micrographs for conductive inks containing as-prepared (**a**,**b**) G6-OH(AuPd)_309_; (**c**,**d**) G6-OH(Au_3_Pd)_309_; and (**e**,**f**) G6-OH(AuPd_3_)_309_ DENs. The yellow arrow on each ac-STEM micrograph corresponds to the x-axis of the EDS line scan. The scale bar is 5.0 nm.

EDS was used to confirm and quantify the alloy compositions of G6-OH(Au_x_Pd_y_)_309_ DENs. Figure 2 shows the EDS line scans and corresponding micrographs for as-prepared G6-OH(Au_x_Pd_y_)_309_ DENs. For example, Figure 2a displays the line scans for the Au and Pd signals for G6-OH(AuPd)_309_ DENs. Figure 2b is a representative micrograph for G6-OH(AuPd)_309_ DENs, where the yellow arrow represents the line on which the EDS signal was collected. The atomic profiles for all compositions of G6-OH(Au_x_Pd_y_)_309_ DENs indicate that the NPs are alloyed [24].

Additionally, Table 1 contains the percentages of Au and Pd extracted from EDS maps of five individual NPs. The key results are that the percentages of Au and Pd quantified by EDS reflect the ratios of Au and Pd used for DEN synthesis, and that G6-OH(Au_x_Pd_y_)_309_ DENs have an alloy structure.

### 3.2. Electrocatalytic EOR Activity of DENs

**Figure 3:**
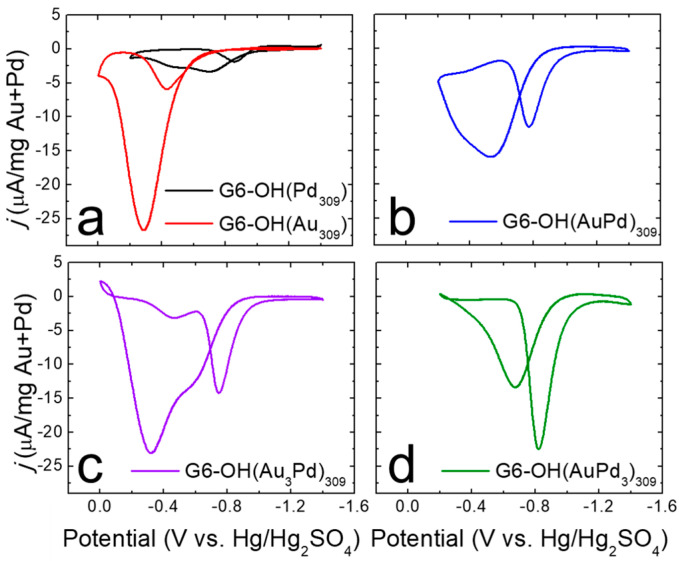
Electrocatalytic EOR CVs for (**a**) G6-OH(Pd_309_) and G6-OH(Au_309_); (**b**) G6-OH(AuPd)_309_; (**c**) G6-OH(Au_3_Pd)_309_; and (**d**) G6-OH(AuPd_3_)_309_ DENs. The CVs were carried out at a scan rate of 50 mV/s in a N_2_-satd., 1.0 M KOH solution containing 0.50 M ethanol. Electrocatalytic EOR CVs were background subtracted using the background CVs shown in Figure S1.

As described in the Experimental Section, G6-OH(Au_309_), G6-OH(Pd_309_), and G6-OH(Au_x_Pd_y_)_309_ DENs were evaluated for the EOR using cyclic voltammetry in N_2_-satd., 1.0 M KOH solution containing 0.50 M ethanol (Figure 3).

The CVs shown in Figure 3 were background subtracted using the CVs in Figure S1 and normalized to the total weight (in mg) of the metal present. Representative background CVs are provided in Figure S1 of the Supplementary Materials. Table 2 contains quantitative values for the EOR onset potential (defined as the potential at which the current density is 10% of the forward peak current density, *j*_f_), the forward peak current density (*j*_f_), and the ratio of the forward to reverse catalytic peaks (*j*_f_/*j*_r_).

Using the CV for G6-OH(AuPd)_309_ DENs (Figure 3b) as an example, the peaks in the CV are identified as follows. When the potential is scanned from −1.4 V to −0.2 V, a peak is observed at ~−0.6 V. This peak is designated as the forward catalytic peak, and corresponds to the direct oxidation of ethanol on the catalyst surface [35]. When the potential sweep is reversed, a peak is present at ~−0.8 V. This peak, defined as the reverse catalytic peak, results from the oxidation of adsorbed intermediates on the catalyst surface, as well as additional oxidation of ethanol [35]. Although the forward and reverse catalytic peak potentials vary somewhat depending on catalyst composition, the interpretation of the peaks is the same for all catalysts.

Figure 3a shows representative electrocatalytic EOR CVs for monometallic G6-OH(Au_309_) (red) and G6-OH(Pd_309_) DENs (black). The value of *j*_f_ for G6-OH(Au_309_) DENs is ~13-fold higher than for G6-OH(Pd_309_). The onset potential for the G6-OH(Pd_309_) DENs, however, occurs at a potential that is 450 mV more negative than that of G6-OH(Au_309_) DENs. An improved EOR catalyst will display both a higher *j*_f_ compared to G6-OH(Pd_309_) DENs, and a more negative onset potential compared to G6-OH(Au_309_) DENs.

Figure 3b–d show representative electrocatalytic EOR CVs for G6-OH(AuPd)_309_, G6-OH(Au_3_Pd)_309_, and G6-OH(AuPd_3_)_309_ DENs, respectively. The CVs reveal three key observations about the EOR activity of G6-OH(Au_x_Pd_y_)_309_ DENs. First, the *j*_f_ values for G6-OH(Au_x_Pd_y_)_309_ DENs are ~5–10-fold higher than the *j*_f_ values for G6-OH(Pd_309_) DENs. The significantly increased *j*_f_ values indicate that the EOR activity is enhanced at the alloy DENs compared to the monometallic Pd DENs.

Second, G6-OH(Au_x_Pd_y_)_309_ DENs exhibit more negative onset potentials for the EOR compared to G6-OH(Au_309_) DENs. For example, the EOR onset potential at G6-OH(Au_3_Pd)_309_ DENs is shifted ~230 mV more to the negative than the onset potential at the monometallic Au DENs. More generally, as the Pd content increases in the alloy DENs, the EOR onset potential shifts to increasingly more negative values compared to G6-OH(Au_309_) DENs.

Third, G6-OH(AuPd)_309_ and G6-OH(Au_3_Pd)_309_ DENs exhibit the same *j*_f_/*j*_r_ ratios, within error, as the Pd-only DENs. This result indicates that the specified alloy DENs have similarly good resistance to poisoning as the monometallic Pd DENs. To summarize, the foregoing results demonstrate that 1–2 nm G6-OH(Au_x_Pd_y_)_309_ DENs exhibit improved EOR activity when compared to either monometallic Au or Pd DENs.

### 3.3. ac-STEM/EDS Characterization of DENs after CVs

Following electrocatalytic EOR CVs, ex-situ ac-STEM and EDS characterization of G6-OH(Au_309_), G6-OH(Pd_309_), and G6-OH(Au_x_Pd_y_)_309_ DENs were carried out. As described in the Experimental Section, TEM grids for post-electrochemical analysis were prepared by gently wiping the lacey carbon-coated TEM grid across the wetted surface of the GCE [36]. TEM grids were dried under N_2_ and subsequently used for analysis.

Quantitative information about the size distributions of DENs following electrocatalytic EOR CVs is shown in Table 1. The ac-STEM micrographs and size-distribution histograms, obtained by sizing 200 randomly selected NPs, for DEN inks following electrocatalytic EOR CVs, are provided in Figure S2. The important result is that the size distributions of DENs following CVs are the same, within error, as for the as-prepared DENs.

**Figure 4:**
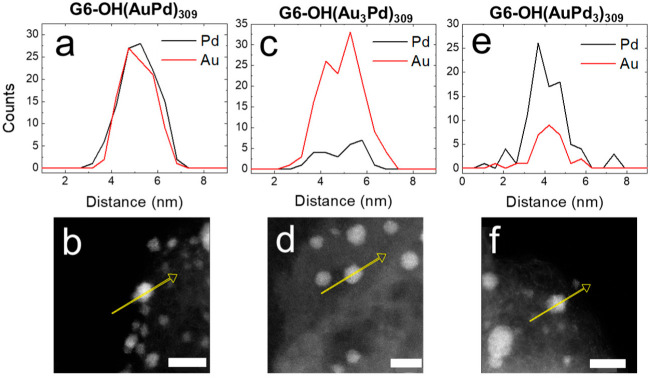
EDS line scans and corresponding ac-STEM micrographs for conductive inks containing (**a**,**b**) G6-OH(AuPd)_309_; (**c**,**d**) G6-OH(Au_3_Pd)_309_; and (**e**,**f**) G6-OH(AuPd_3_)_309_ DENs following EOR CVs. The CVs were carried out at a scan rate of 50 mV/s in a N_2_-satd., 1.0 M KOH solution containing 0.50 M ethanol. The yellow arrow on each ac-STEM micrograph corresponds to the x-axis of the EDS line scan. The scale bar is 5.0 nm.

EDS was used to quantify the compositions of G6-OH(Au_x_Pd_y_)_309_ DENs following electrocatalytic EOR CVs. Figure 4 shows the EDS line scans and corresponding micrographs for G6-OH(Au_x_Pd_y_)_309_ DENs following EOR CVs.

The EDS line scans for all compositions of G6-OH(Au_x_Pd_y_)_309_ DENs indicate that the NPs retain their initial alloy compositions following CVs. Additionally, Table 1 contains the quantitative percentages of Au and Pd following EOR CVs extracted from EDS maps of five individual NPs. Although small changes in Au and Pd composition occur following CVs, the percent compositions of Au and Pd are the same, within error, as for the as-prepared DENs. The key result from this section is that electrocatalytic EOR CVs do not significantly change the size or composition of 1–2 nm DENs.

### 3.4. Electrocatalytic EOR Chronoamperograms (CAs)

For the electrocatalytic EOR, catalyst stability is usually assessed using chronoamperometry [5,6,8,9,37]. Specifically, the potential of the electrode is typically held at the peak potential of the preceding electrocatalytic CV for times ranging from 30 min to 12 h. In the present case, 1.0 h CAs for G6-OH(Au_309_), G6-OH(Pd_309_), and G6-OH(Au_x_Pd_y_)_309_ DENs were carried out in N_2_-satd., 1.0 M KOH containing 0.50 M ethanol. The electrode was held at the peak potential for each catalyst.

**Figure 5:**
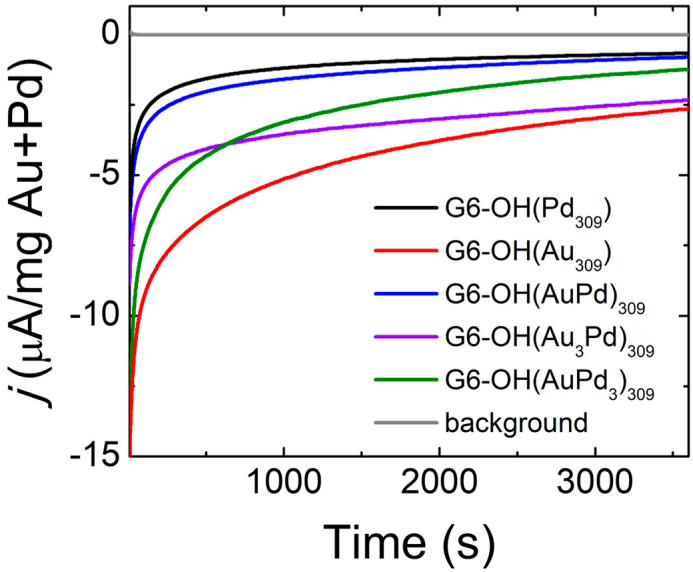
Electrocatalytic EOR CAs for the indicated DENs. The CAs were carried out in a N_2_-satd., 1.0 M KOH solution containing 0.50 M ethanol, by holding the electrode potential for 1.0 h at the peak potential of the forward scan of the EOR CV. For clarity, the first 10 s of the CA are not shown in the figure.

Figure 5 presents the electrocatalytic EOR CAs for the DENs in this study. For clarity, the first 10 s of the CAs, where an initial sharp decrease in *j* occurs, are not shown.

Qualitatively, the CA data exhibit a current decay for all the DEN catalysts. Similar results from others have been interpreted to mean that NP catalysts are electrochemically stable [8,9,38]. This interpretation seems odd given the decaying current, but perhaps the authors of these earlier studies meant that because the current does not go to zero the catalysts are stable. Our own interpretation is that EOR CAs such as these do not provide much insight into changes in catalyst size or structure that might occur during prolonged electrochemical measurements [13]. One final point is worth mentioning. The authors of these previous studies indicated (sometimes by omission) that the CAs were obtained in quiescent solutions. For comparative purposes, we followed this precedent for the data in Figure 5. As a control, however, we obtained a set of CAs that were carried out with stirring (Figure S3). There are no significant differences between the stirred and unstirred CAs.

CVs were collected for each DEN composition both before and after the 1.0 h CA measurement (Figure S4). The results are shown in Table 2, which provides quantitative values for the EOR onset potential, *j*_f_, and *j*_f_/*j*_r_. The values shown in Table 2 indicate that the 1.0 h CAs do not induce significant changes in the electrocatalytic onset potential or *j*_f_/*j*_r_ for the DEN catalysts. Significant decreases in *j*_f_, however, are observed for the G6-OH(AuPd_3_)_309_ and G6-OH(Au_309_) DENs. For the G6-OH(Au_309_) DENs, *j*_f_ decreases by ~34% compared to the as-prepared Au DENs. The decrease in *j*_f_ will be discussed in the next section.

### 3.5. ac-STEM/EDS Characterization of DENs after CAs

Following the 1.0 h electrocatalytic EOR CAs described in the previous section, the DENs were characterized using ex-situ ac-STEM and EDS. As described in the Experimental Section, TEM grids for ex-situ analysis were prepared by gently wiping the lacey carbon-coated TEM grid across the wetted surface of the GCE and drying under N_2_.

**Figure 6:**
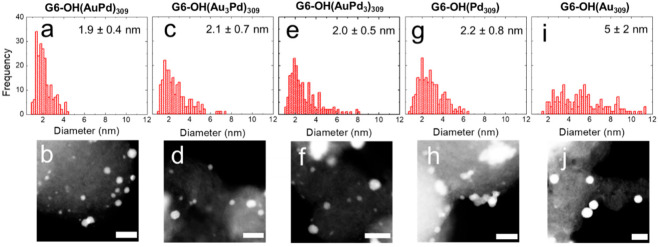
Size-distribution histograms and ac-STEM micrographs for conductive inks containing (**a**,**b**) G6-OH(AuPd)_309_; (**c**,**d**) G6-OH(Au_3_Pd)_309_; (**e**,**f**) G6-OH(AuPd_3_)_309_; (**g**,**h**) G6-OH(Pd_309_); and (**i**,**j**) G6-OH(Au_309_) DENs after electrocatalytic EOR CAs. CAs were carried out in a N_2_-satd., 1.0 M KOH solution containing 0.50 M ethanol, by holding the electrode potential for 1.0 h at the peak potential of the forward scan of the EOR CV. The scale bar is 10.0 nm.

Figure 6 displays ac-STEM micrographs and size-distribution histograms, obtained by sizing 200 randomly selected NPs, for DEN inks following 1.0 h electrocatalytic EOR CAs.

Table 1 provides quantitative information extracted from these data. The size distributions reveal two key results. First, within error, the average sizes of G6-OH(Au_x_Pd_y_)_309_ and G6-OH(Pd_309_) DENs do not change following the 1.0 h CAs. Second, a significant increase is observed in the size and polydispersity of monometallic Au DENs (from 2.5 ± 0.4 nm following CVs, to 5 ± 2 nm) following the 1.0 h CA. Increased NP size and polydispersity, as is observed for G6-OH(Au_309_) DENs, likely leads to the decreases in *j*_f_ values discussed in the previous section [13]. These results demonstrate that G6-OH(Au_x_Pd_y_)_309_ and G6-OH(Pd_309_) DENs are stable in size during 1.0 h CAs, particularly compared to monometallic Au DENs.

**Figure 7:**
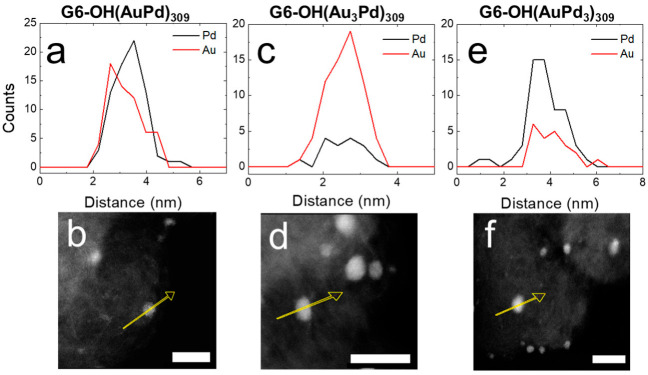
EDS line scans and corresponding ac-STEM micrographs for conductive inks containing (**a**,**b**) G6-OH(AuPd)_309_; (**c**,**d**) G6-OH(Au_3_Pd)_309_; and (**e**,**f**) G6-OH(AuPd_3_)_309_ DENs after a 1.0 h EOR CA. CAs were carried out in a N_2_-satd., 1.0 M KOH solution containing 0.50 M ethanol, by holding the electrode potential for 1.0 h at the peak potential of the forward scan of the EOR CV. The yellow arrow on each ac-STEM micrograph corresponds to the x-axis of the EDS line scan. The scale bar is 5.0 nm.

Figure 7 shows EDS line scans and corresponding micrographs for G6-OH(Au_x_Pd_y_)_309_ DENs following 1.0 h electrocatalytic CAs. The similarity of the atomic profiles for all compositions of the G6-OH(Au_x_Pd_y_)_309_ DENs indicate that they retain their alloy character following 1.0 h CAs.

Table 1 shows the percentages of Au and Pd extracted from EDS maps of five individual NPs. Within error, the Au and Pd percentages do not change significantly from those observed prior to the 1.0 h CAs. In addition to the 1.0 h EOR CAs, longer 2.0 h CAs were also carried out for the G6-OH(Au_x_Pd_y_)_309_ DENs which were subsequently characterized using ac-STEM and EDS. Within error, the average sizes and compositions of the G6-OH(Au_x_Pd_y_)_309_ DENs are the same for the 1.0 and 2.0 h CAs (Table S1).

There are two key results discussed in this section. First, following 1.0 or 2.0 h CAs, the sizes of G6-OH(Pd_309_) and G6-OH(Au_x_Pd_y_)_309_ DENs do not change significantly, while the size and polydispersity of G6-OH(Au_309_) DENs do change. Second, the structure and composition of G6-OH(Au_x_Pd_y_)_309_ DENs do not change significantly following 1.0 or 2.0 h CAs.

## 4. Summary and Conclusions

The electrocatalytic EOR activity of 1–2 nm G6-OH(Au_x_Pd_y_)_309_, G6-OH(Au_309_), and G6-OH(Pd_309_) DENs was evaluated using cyclic voltammetry and chronoamperometry. Following electrocatalysis, ac-STEM and EDS were used to characterize DEN size and composition. Two main findings emerge from this study. First, alloyed AuPd DENs demonstrate enhanced electrocatalytic EOR activity compared to either monometallic Au or Pd DENs. Specifically, G6-OH(AuPd)_309_ and G6-OH(Au_3_Pd)_309_ DENs exhibit higher peak current densities compared to Pd DENs and more negative onset potentials compared to Au DENs. Second, ac-STEM and EDS characterization reveal that the size and composition of G6-OH(Au_x_Pd_y_)_309_ and G6-OH(Pd_309_) DENs do not change significantly following 1.0 or 2.0 h electrocatalytic EOR CAs, while monometallic Au DENs significantly increase in size and polydispersity under the same conditions.

Notably, we used ac-STEM and EDS to characterize the size and composition of 1–2 nm DENs before and after EOR electrocatalysis. NP stability during the electrocatalytic EOR is typically determined using chronoamperometry. The resulting CAs, however, do not provide insight into changes in catalyst size, structure, or composition that may occur during electrocatalysis, which is particularly problematic when experimental measurements are correlated to theory [23].

## Figures and Tables

**Table 1:** Size distributions and Au/Pd compositions of DENs both as prepared and after electrocatalytic EOR measurements. Size distributions were obtained from 200 NPs. The atomic percentages of Au and Pd were extracted from EDS maps of 5 individual NPs.

	As Prepared	After EOR CVs	After 1.0 h EOR CA	As Prepared		After EOR CVs		After 1.0 h EOR CA	
	nm	nm	nm	Au %	Pd %	Au %	Pd %	Au %	Pd %
G6-OH(AuPd)_309_	2.0 ± 0.5	1.9 ± 0.4	1.9 ± 0.4	47 ± 7	53 ± 7	55 ± 6	45 ± 6	65 ± 6	35 ± 6
G6-OH(Au_3_Pd)_309_	1.8 ± 0.3	1.9 ± 0.3	2.1 ± 0.7	77 ± 7	23 ± 7	81 ± 7	19 ± 7	84 ± 7	16 ± 7
G6-OH(AuPd_3_)_309_	1.6 ± 0.3	1.7 ± 0.4	2.0 ± 0.5	31 ± 6	69 ± 6	31 ± 6	69 ± 6	40 ± 5	60 ± 5
G6-OH(Pd_309_)	1.8 ± 0.3	1.8 ± 0.3	2.2 ± 0.8	-	100	-	100	-	100
G6-OH(Au_309_)	2.3 ± 0.4	2.5 ± 0.4	5 ± 2	100	-	100	-	100	-

**Table 2:** Results extracted from electrocatalytic EOR CVs for DEN catalysts both before and after a 1.0 h CA. The first three columns correspond to the values for CVs collected before the 1.0 h CA. The first column provides the EOR onset potential (defined as the potential at which the current density is 10% of the forward peak current density, *j*_f_). The second column lists the forward peak current density, *j*_f_. The third column shows the ratio of the forward to reverse catalytic peak current densities (*j*_f_/*j*_r_). The fourth, fifth, and sixth columns provide the same parameters, but for CVs collected following a 1.0 h CA. CVs were carried out at a scan rate of 50 mV/s in a N_2_-satd., 1.0 M KOH solution containing 0.50 M ethanol and background subtracted using the background CVs in Figure S1. Standard deviations are based on three independent experiments.

	Before 1.0 h CA			After 1.0 h CA		
	Onset Potential (V)	Peak Current Density, *j*_f_ (µA/mg Au + Pd)	*j*_f_ */j*_r_	Onset Potential (V)	Peak Current Density, *j*_f_ (µA/mg Au + Pd)	*j*_f_/*j*_r_
G6-OH(AuPd)_309_	−0.82 ± 0.01	16.2 ± 0.2	1.4 ± 0.1	−0.85 ± 0.01	14.1 ± 0.5	1.5 ± 0.2
G6-OH(Au_3_Pd)_309_	−0.79 ± 0.03	25 ± 2	2.1 ± 0.7	−0.76 ± 0.01	22 ± 1	1.3 ± 0.6
G6-OH(AuPd_3_)_309_	−0.91 ± 0.01	15 ± 1	0.56 ± 0.03	−0.88 ± 0.03	8 ± 3	0.3 ± 0.1
G6-OH(Pd_309_)	−1.03 ± 0.01	3.2 ± 0.3	1.7 ± 0.2	−0.97 ± 0.03	2.8 ± 0.3	1.5 ± 0.2
G6-OH(Au_309_)	−0.56 ± 0.04	29 ± 3	4.8 ± 0.3	−0.51 ± 0.03	19 ±5	4.5 ± 0.2

## Data Availability

The data are available on reasonable request from the corresponding author.

## Supplementary Materials

The following supporting information can be downloaded at: https://www.mdpi.com/article/10.3390/nano12224093/s1, Figure S1: Representative background CVs in 1.0 M KOH; Figure S2: ac-STEM micrographs and size-distribution histograms of DENs after EOR CVs; Figure S3: Electrocatalytic EOR CAs in stirred solution; Figure S4: Electrocatalytic EOR CVs collected before and after 1.0 h EOR CA; Figure S5: ac-STEM micrographs and size-distribution histograms of G6-OH(AuxPdy)309 DENs after 2.0 h EOR CA; Figure S6: EDS line scans and micrographs for G6-OH(AuxPdy)309 DENs after 2.0 h EOR CA; Table S1: Size distributions and Au/Pd compositions of G6-OH(AuxPdy)309 DENs after 2.0 h EOR CA.
