# Supplementary material for: Ethanol Electrooxidation at 1–2 nm AuPd Nanoparticles
Source: Nanomaterials (Basel). 2022 Nov 21;12(22):4093. doi: 10.3390/nano12224093 (PMC9692959; doi:10.3390/nano12224093)
Supplement: Supplementary file 1 [file nanomaterials-12-04093-s001.zip › nanomaterials-2030036-supplementary.pdf]

Supporting Information

Ethanol Electrooxidation at 1–2 nm AuPd Nanoparticles

Juliette W. Strasser and Richard M. Crooks *

Department of Chemistry and Texas Materials Institute, The University of Texas at Austin, 2506 Speedway, Stop A5300, Austin, TX 78712-1224, USA; jstrasser@utexas.edu
* Correspondence: crooks@cm.utexas.edu; Tel.: +1-512-475-8674

Representative background CVs in 1.0 M KOH, Figure S1.

ac-STEM micrographs and size-distribution histograms of DENs after EOR CVs, Figure S2.

Electrocatalytic EOR CAs in stirred solution, Figure S3.

Electrocatalytic EOR CVs collected before and after 1.0 h EOR CA, Figure S4.

ac-STEM micrographs and size-distribution histograms of G6-OH(Au_xPd_y)$_{309}$ DENs after 2.0 h EOR CA, Figure S5.

EDS line scans and micrographs for G6-OH(Au_xPd_y)$_{309}$ DENs after 2.0 h EOR CA, Figure S6.

Size distributions and Au/Pd compositions of G6-OH(Au_xPd_y)$_{309}$ DENs after 2.0 h EOR CA, Table S1.

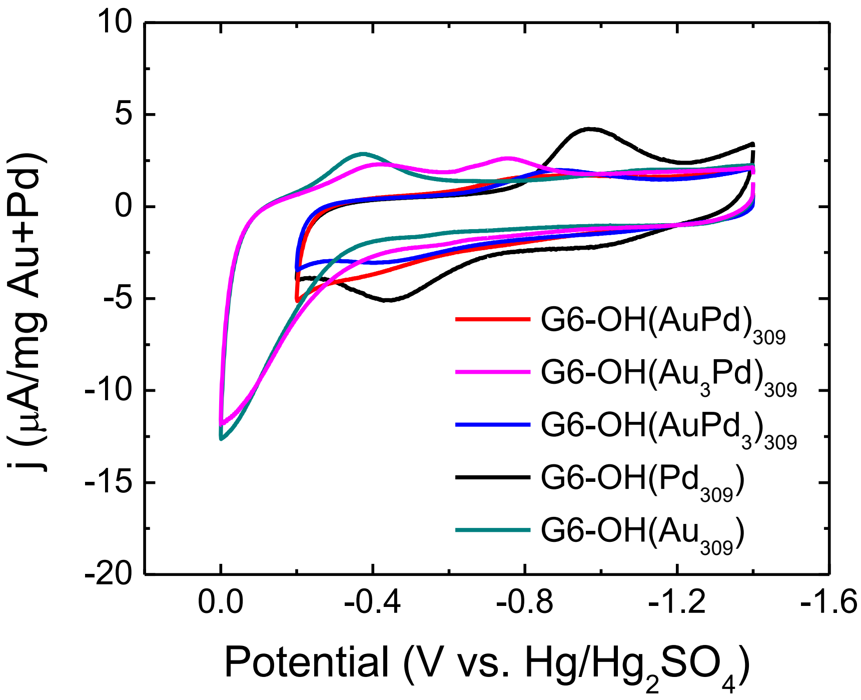

Figure S1. Representative background CVs for the indicated DEN compositions. CVs were collected at a scan rate of 50 mV/s in N_2-satd., 1.0 M KOH and used for background subtraction from the electrocatalytic EOR CVs shown in Figure 3 of the main text and Figure S4.

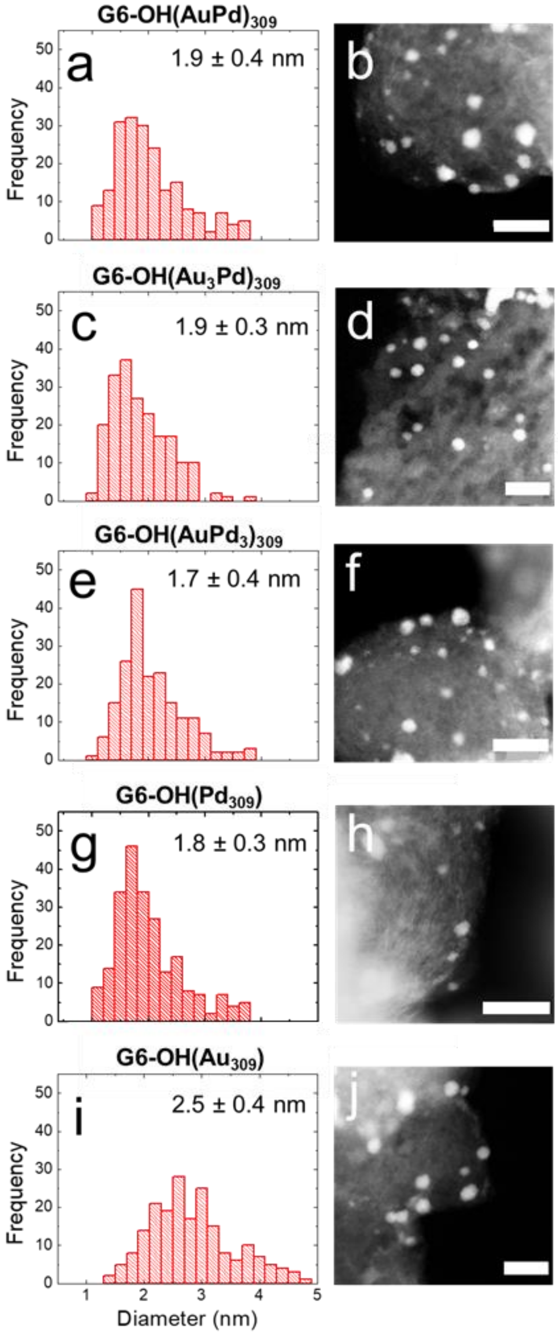

Figure S2. ac-STEM micrographs and corresponding size-distribution histograms for conductive inks containing (a, b) G6-OH(AuPd)$_{309}$; (c, d) G6-OH(Au$_3$Pd)$_{309}$; (e, f) G6-OH(AuPd$_3$)$_{309}$; (g, h) G6-OH(Pd$_{309}$); and (i, j) G6-OH(Au$_{309}$) DENs after electrocatalytic EOR CVs. CVs were carried out at a scan rate of 50 mV/s in a N_2-satd., 1.0 M KOH solution containing 0.50 M ethanol. The scale bar is 10.0 nm. Within the indicated error, the average sizes of the indicated DENs do not change significantly following EOR CVs.

.

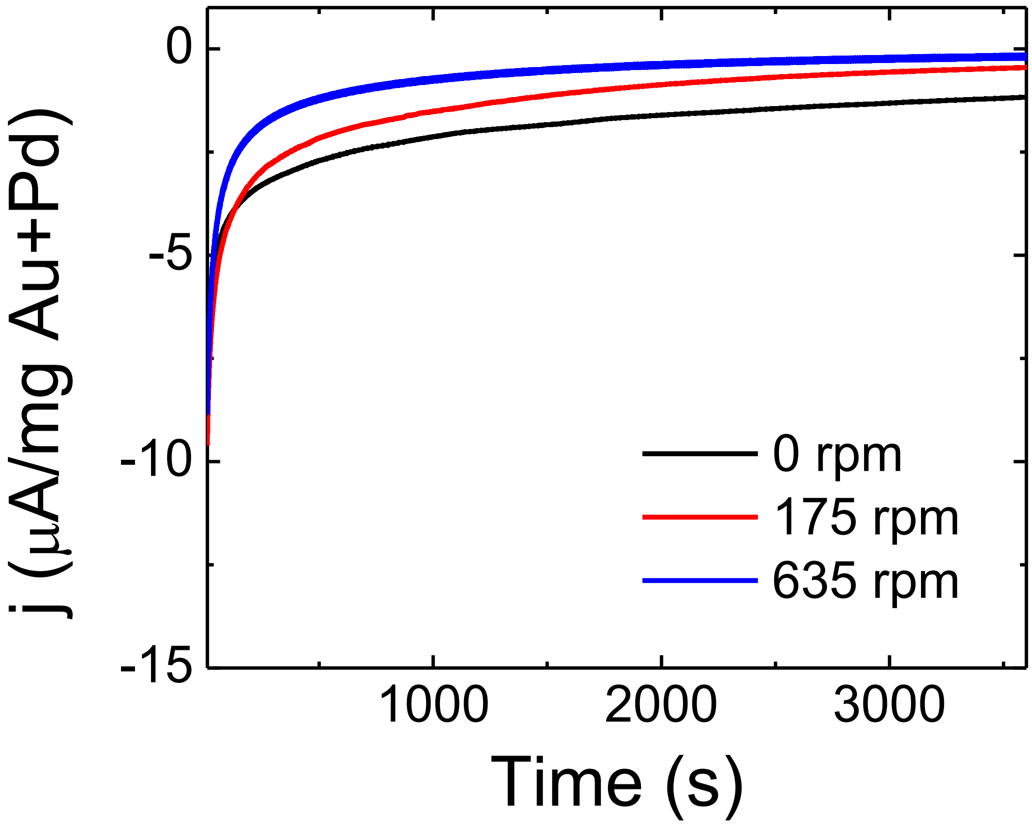

Figure S3. Electrocatalytic EOR CAs for G6-OH(AuPd)$_{309}$ DENs, stirred at 0, 175, and 635 rpm. The CAs were carried out in a N_2-satd., 1.0 M KOH solution containing 0.50 M ethanol, by holding the electrode potential for 1.0 h at the peak potential of the forward scan of the EOR CV. The solution was stirred using a stir bar and stir plate. For clarity, the first 10 s of the CAs, where an initial sharp decrease in *j* occurs, are not shown.

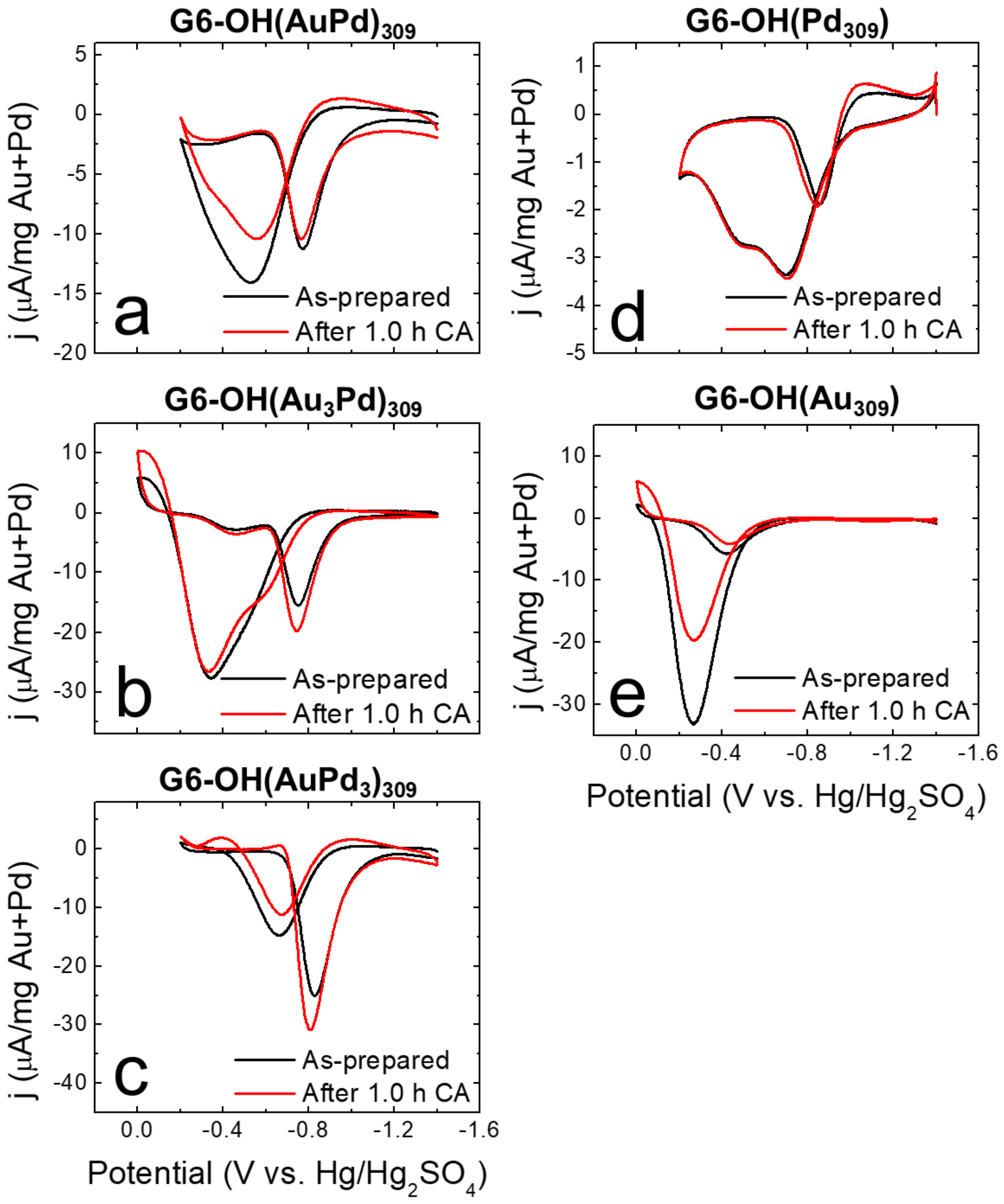

Figure S4. CVs obtained before and after a 1.0 h EOR CA for (a) G6-OH(AuPd)309; (b) G6-OH(Au3Pd)309; (c) G6-OH(AuPd3)309; (d) G6-OH(Pd309); and (e) G6-OH(Au309) DENs. The CVs were carried out at a scan rate of 50 mV/s in N_2-satd., 1.0 M KOH containing 0.50 M ethanol and background subtracted using the CVs in Figure S1. In most cases there are only minor differences between the CVs collected before and after the 1.0 h CA.

.

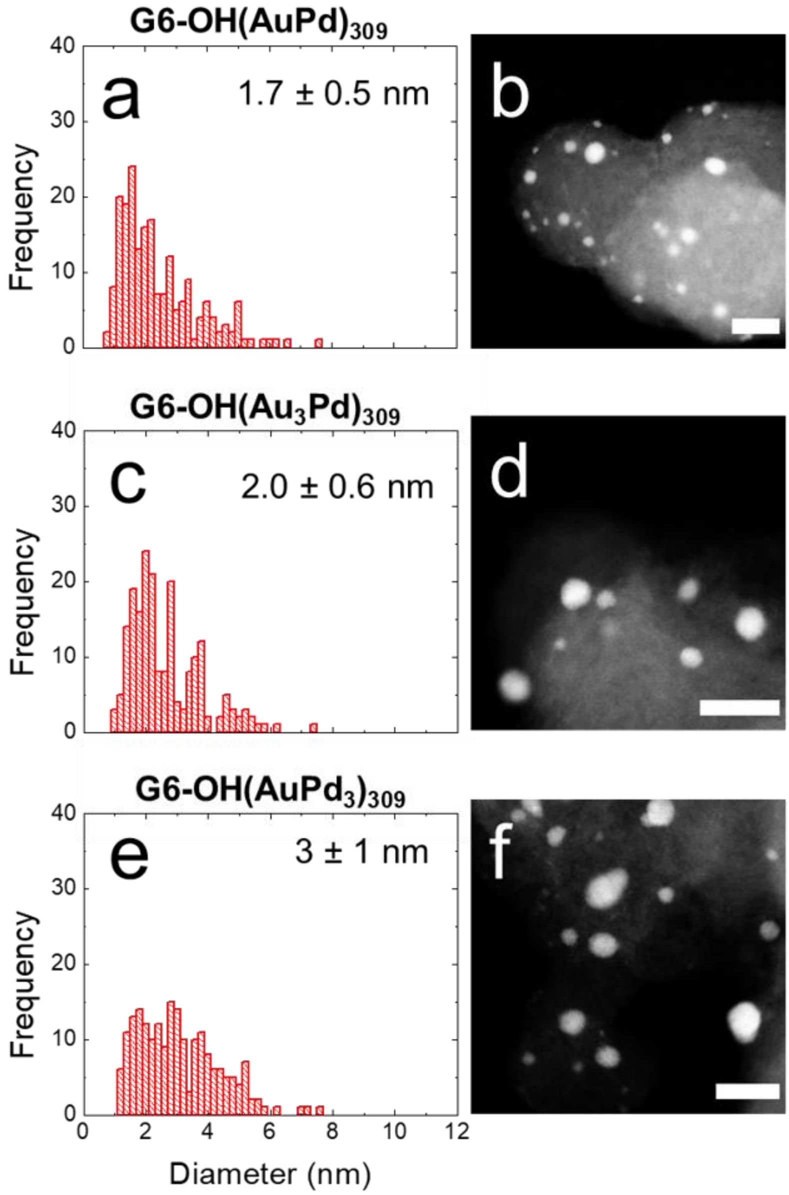

Figure S5. ac-STEM micrographs and corresponding size-distribution histograms for (a, b) G6-OH(AuPd)$_{309}$; (c, d) G6-OH(Au$_3$Pd)$_{309}$; and (e, f) G6-OH(AuPd$_3$)$_{309}$ DENs following a 2.0 h EOR CA. CAs were carried out in a N_2-satd., 1.0 M KOH solution containing 0.50 M ethanol by holding the electrode potential for 2.0 h at the peak potential of the forward scan of the EOR CV. The scale bar is 10.0 nm. Within the indicated error, the average sizes of G6-OH(Au$_x$Pd$_y$)$_{309}$ DENs do not change significantly between the 1.0 and 2.0 h CAs.

.

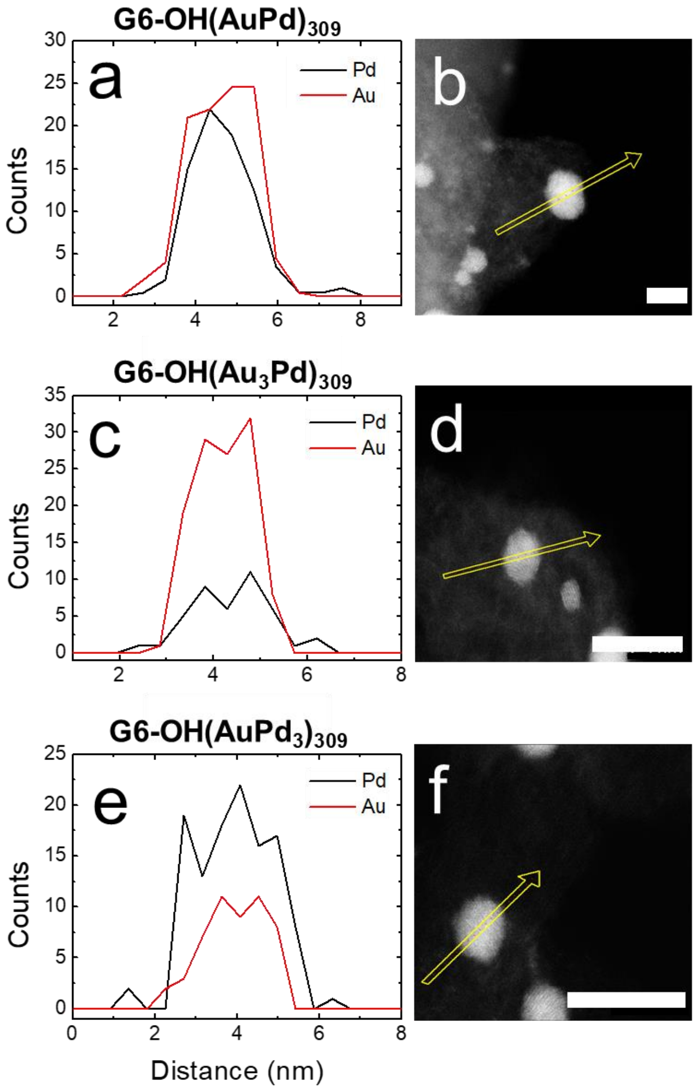

Figure S6. EDS line scans and corresponding micrographs for (a, b) G6-OH$(AuPd)_{309}$; (c, d) G6-OH$(Au_3Pd)_{309}$; and (e, f) G6-OH$(AuPd_3)_{309}$ DENs following a 2.0 h CA. CAs were carried out in a N_2-satd., 1.0 M KOH solution containing 0.50 M ethanol, by holding the electrode potential for 2.0 h at the peak potential of the forward scan of the EOR CV. The yellow arrow on each ac-STEM micrograph corresponds to the *x*-axis of the EDS line scan. The scale bar is 5.0 nm. The similarity of the atomic profiles for all compositions of G6-OH$(Au_xPd_y)_{309}$ DENs indicate that they retain their alloy compositions following the 2.0 h CA.

.

Table S1. Size distributions and Au/Pd compositions of DENs after 2.0 h electrocatalytic EOR CAs. CAs were carried out in a N_2-satd., 1.0 M KOH solution containing 0.50 M ethanol, by holding the electrode potential for 2.0 h at the peak potential of the forward scan of the EOR CV. Size distributions were obtained from 200 NPs. The atomic percentages of Au and Pd were extracted from the EDS maps of 5 individual NPs. Within error, the average sizes and compositions of the G6-OH(Au_xPd_y)$_{309}$ DENs do not change significantly between the 1.0 and 2.0 h CAs.

	**Diameter (nm)**	**% Au**	**% Pd**
G6-OH(AuPd)$_{309}$	1.7 ± 0.5	72 ± 4	27 ± 4
G6-OH(Au_3Pd)$_{309}$	2.0 ± 0.6	92 ± 3	8 ± 3
G6-OH($AuPd_3$)$_{309}$	3 ± 1	40 ± 5	60 ± 5
